# Disentangling anthropogenic and dynamic contributions to recent ocean warming

**DOI:** 10.1038/s41612-025-01043-7

**Published:** 2025-04-26

**Authors:** Jiheun Lee, Rémi Tailleux, Till Kuhlbrodt

**Affiliations:** 1 Department of Meteorology, University of Reading, Reading, UK; 2 National Centre for Atmospheric Science, Reading, UK

**Keywords:** Ocean sciences, Physical oceanography, Climate sciences, Climate change, Ocean sciences

## Abstract

As the ocean absorbs over 90% of excess radiative heat, recent ocean warming is shaped by a combination of anthropogenic surface heat gain and dynamical processes redistributing heat. To distinguish these contributions, we introduce a novel framework that decomposes temperature changes into three components: ‘spice’ (density-compensated variability) and ‘heave’ (density-contributing variability), with heave further divided into ‘passive’ (net warming) and ‘dynamic’ (redistribution) contributions. Passive heave captures anthropogenic warming subducted along isopycnals, while spice and dynamic heave, which globally sum to zero, represent heat redistribution. Observations and climate models demonstrate general agreement on passive heave, establishing it as a key oceanic fingerprint of anthropogenic climate change. In contrast, dynamic heave, driven by interannual-to-decadal variability, exhibits significant spatial heterogeneity, with notable discrepancies between models and observations. This framework links ocean heat uptake to sea-level change, with passive heave driving global thermosteric rise and dynamic heave contributing to regional dynamic sea level changes.

## Introduction

Earth’s climate system has undergone significant warming over recent decades, driven by increasing emissions of atmospheric greenhouse gases. The ocean has absorbed the vast majority of this excess heat, with the upper 2.5 m alone holding an equivalent heat capacity to the entire atmosphere. This heat uptake has led to the thermal expansion of sea water, making it a major contributor to global mean sea-level rise^1,2^. However, understanding and quantifying ocean heat uptake remains challenging due to its complex interaction with natural climate variability. Identifying externally forced warming is often complicated by climate variability that significantly alters ocean dynamics, such as the El Niño-Southern Oscillation (ENSO), North Atlantic Oscillation (NAO), wind stress variations and fluctuations in air-sea heat and freshwater fluxes^3–5^. A notable example is how internally generated decadal variability, such as La Niña-like conditions, temporarily counterbalanced greenhouse gas-induced warming in the Pacific during the early 2000s^6,7^. This raises a fundamental question: to what extent does observed ocean warming reflect direct anthropogenic influences versus being masked or enhanced by climate variability?

To address this question, considerable efforts have been made to understand how anthropogenic warming signals penetrate the ocean interior. It is generally postulated that heat anomalies, once subducted into the thermocline within subtropical gyres, behave analogously to passive tracers, being transported along isopycnals by background ocean circulation^8,9^. Building on this idea, ref. ^10^ introduced a framework using numerical models to decompose ocean heat uptake into two components: added heat (introduced via altered air-sea fluxes and passively distributed) and redistributed heat (driven by changes in ocean circulation). This framework has been widely adopted and applied in various experimental approaches, providing insights into passive ocean heat uptake shaped by the transport of surface temperature anomalies under climatological circulation^11–17^. In the real ocean, however, such heat addition is not necessarily density-compensated; rather, it inherently modifies the density field along its pathway, often leading to lighter density classes. Its assumed passive character is therefore fundamentally distinct from that of density-compensated ‘spice’ anomalies, which calls for a more rigorous dynamical framework to account for how heat uptake can manifest as passive in the ocean.

To move beyond the reliance on imposed surface boundary conditions in the passive temperature tracer approach, ref. ^18^ proposed a framework based on water mass transformation and optimal transportation theory to quantify material and redistributed contributions to ocean heat content change. However, this approach still involves subjective choices, particularly in defining a distance metric in temperature-salinity space. Here, we present a robust framework for diagnosing passive heat uptake by introducing a novel water mass-based method that refines the traditional spice/heave decomposition of temperature changes^8,9,19,20^. Our method distinguishes between ‘spice’ (density-compensated thermohaline variability behaving like passive tracers) and ‘heave’ anomalies (apparent temperature changes related to vertical displacements of isopycnal surfaces). In the context of global warming, we emphasise that heave can result from diabatic as well as adiabatic processes, as evidenced by the progressive deepening of mid-thermocline isopycnals^21–24^. The need to separate diabatic and adiabatic heave has been previously recognised–for example, by ref. ^25^, who introduced the concepts of external and internal heaving modes to distinguish between the two. However, despite its importance for accurately characterising the dynamic drivers of ocean warming, achieving such a decomposition has remained an unresolved challenge.

Central to our framework is a time-dependent labelling system for density surfaces, denoted as *ℓ*(*γ*, *t*), where *γ* = *γ*(*S*, *θ*) represents the quasi-material density variable defining isopycnal surfaces. By constraining *ℓ*(*γ*, *t*) to vary temporally only under diabatic modifications of the stratification, we can identify diabatic warming along constant iso-*ℓ* surfaces as a passive process, while capturing adiabatic heave through the time-independent component of *ℓ*(*γ*, *t*). This approach offers two key advances: it provides a clearer mechanism for tracking anthropogenic anomalous warming in the ocean interior as a passive process, while simultaneously distinguishing it from the redistribution of existing heat through dynamic processes. In this study, we directly evaluate the signatures of recent ocean warming linked to anthropogenic heat uptake and dynamic heat redistribution, with the goal of improving future projections of ocean warming patterns and sea-level changes over the coming century.

## Results

### Dynamic and passive aspects of spice and heave

We revisit the classical decomposition of temperature anomalies into spice and heave components, aiming to establish a clearer physical basis for distinguishing between their dynamically active and passive contributions. In oceanography, passive tracers are those that do not directly influence ocean dynamics, while active tracers, such as temperature and salinity, affect density and thus play a role in driving ocean circulation changes. Although temperature and salinity are inherently active tracers, certain aspects of their variability can be considered passive. To isolate these passive components, we develop a theoretical framework that provides a precise definition of ‘passive’ in this context.

To analyse the contributions of temperature and salinity to density, we assume the existence of a suitable quasi-material density variable, *γ*(*S*, *θ*). Using this framework, temperature *θ*(**x**, *t*) and salinity *S*(**x**, *t*) at position **x** and time *t* can be separated into two components:

1 $$\theta ({\bf{x}},t)={\theta }_{0}(\gamma )+{\theta }^{{\prime} }({\bf{x}},t),\qquad S({\bf{x}},t)={S}_{0}(\gamma )+{S}^{{\prime} }({\bf{x}},t).$$

Here, *θ*_0_(*γ*) and *S*_0_(*γ*) represent the components of *θ* and *S* that directly contribute to density, while ${\theta }^{{\prime} }$ and ${S}^{{\prime} }$ compensate for density changes. The former components satisfy the following relationships:

2 $$\gamma ({S}_{0}({\gamma }_{0}),{\theta }_{0}({\gamma }_{0}))={\gamma }_{0},$$

while ${\theta }^{{\prime} }$ and ${S}^{{\prime} }$ represent the *γ*-compensated parts:

3 $$\begin{array}{l}\gamma ({S}_{0}({\gamma }_{0})+{S}^{{\prime} },{\theta }_{0}({\gamma }_{0})+{\theta }^{{\prime} })=\gamma ({S}_{0}({\gamma }_{0}),{\theta }_{0}({\gamma }_{0}))\\ \Rightarrow \qquad {\gamma }_{S}({S}_{0},{\theta }_{0}){S}^{{\prime} }+{\gamma }_{\theta }({S}_{0},{\theta }_{0}){\theta }^{{\prime} }\approx 0.\end{array}$$

Taking the Eulerian time derivative of Eq. (1) yields the following decomposition of temperature changes:

4 $$\frac{\partial \theta }{\partial t}=\frac{d{\theta }_{0}}{d\gamma }(\gamma )\frac{\partial \gamma }{\partial t}+\frac{\partial {\theta }^{{\prime} }}{\partial t}({\bf{x}},t).$$

The first term represents the apparent temperature changes related to the vertical displacements of density surfaces, a process known as ‘heave’. The second term captures temperature changes that do not affect density, referred to as ‘spice’. To determine whether these components are dynamically active or passive, we consider their impact on in-situ density *ρ*:

5 $$\rho ={\rho }_{0}(z,t)+{\rho }^{{\prime} }({\bf{x}},t),$$

where *ρ* is decomposed as the sum of a one-dimensional function of depth and time, *ρ*_0_(*z*, *t*), and a perturbation, ${\rho }^{{\prime} }({\bf{x}},t)$. To ensure that *ρ*_0_(*z*, *t*) depends on time only in the presence of diabatic sources or sinks of heat and salt, it is defined through an adiabatic and isohaline rearrangement of fluid parcels that minimises potential energy, as is classically done in standard Lorenz available potential energy theory^26,27^. Consequently, the use of Lorenz reference state *ρ*_0_(*z*, *t*) naturally separates diabatic and adiabatic effects^28^. Based on Eq. (5), temperature anomalies can be considered passive in two cases: when they do not affect density at all, or when they only affect the background density profile *ρ*_0_(*z*, *t*) without altering the perturbation ${\rho }^{{\prime} }$. In both cases, the anomalies do not create horizontal density gradients that influence ocean circulation. Based on this reasoning, the spice component in Eq. (4) is primarily passive, as it represents density-compensated changes. Heave, on the other hand, is typically considered active because it involves vertical displacements that affect density gradients and drive ocean dynamics. However, it can also have a passive component when these displacements are solely associated with *ρ*_0_(*z*, *t*), which results from diabatic processes such as heat addition due to Earth’s energy imbalance. In traditional analyses, the dynamic and passive components have often been combined into a single ‘heave’ category, which obscures a more nuanced understanding of their respective roles in thermohaline variability.

**Fig. 1:**
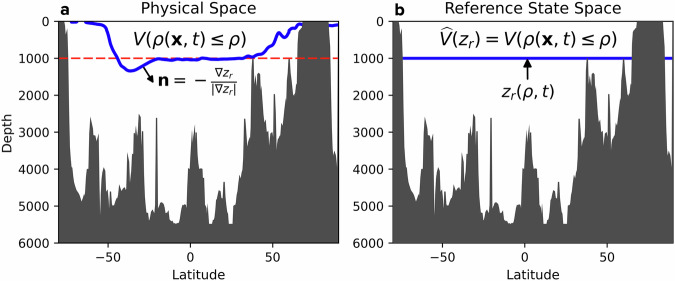
Schematics illustrating the physical principles underlying the construction of the *z*_*r*_ surfaces for a Boussinesq ocean. Adiabatic and isohaline sorting of water parcels from **a** physical space results in a stably stratified reference state in (**b**). The reference depth *z*_*r*_(*ρ*, *t*) is defined so that the volume of water comprised of all fluid parcels with density less than *ρ* in (**a**) matches the same volume of water between *z* = 0 and *z* = *z*_*r*_ in (**b**) of the reference state space. In a compressible ocean, *z*_*r*_ becomes a function of (*S*, *θ*, *t*) rather than (*ρ*, *t*). Schematic adapted from Fig. 2 of ref. ^51^).

The Lorenz reference density profile *ρ*_0_(*z*, *t*) can serve to define a reference position *z*_*r*_(*S*, *θ*, *t*) for fluid parcels as a solution to the equation:

6 $$\rho [S,\theta ,{p}_{0}({z}_{r},t)]={\rho }_{0}({z}_{r},t),$$

where *p*_0_(*z*, *t*) is such that *gρ*_0_(*z*, *t*) = −*dp*_0_/*dz*(*z*, *t*)^29^. Physically, Eq. (6) states that *z*_*r*_ represents the level at which a fluid parcel becomes neutrally buoyant relative to *ρ*_0_(*z*, *t*). By design, *z*_*r*_ is a quasi-material variable, a function of (*S*, *θ*) that also depends on time whenever *ρ*_0_(*z*, *t*) changes due to diabatic modification. Its physical interpretation is illustrated in Fig. 1. The use of *z*_*r*_ in the decomposition enables the definition of density surfaces that are accurately neutral in most of the ocean interior^30^ and provides an objective representation of the dynamic nature of isopycnal surfaces^31^. Furthermore, because *z*_*r*_ has the dimensions of depth with fixed bounds, it is easier to interpret than density, which has varying bounds under diabatic influences. In contrast, conventional density-based decompositions can be often inexact, especially in regions with strong air-sea interactions or in a warming climate where new, lighter density classes emerge near the surface. For these reasons, we opt to use *z*_*r*_ in place of *γ* in Eq. (4).

To isolate diabatic and adiabatic effects, we now reformulate Eq. (1) and Eq. (4) by replacing density *γ* by *z*_*r*_ as follows:

7 $$\theta ({\bf{x}},t)={\theta }_{0}({z}_{r},t)+{\theta }^{{\prime} }({\bf{x}},t),\qquad S({\bf{x}},t)={S}_{0}({z}_{r},t)+{S}^{{\prime} }({\bf{x}},t),$$

8 $$\frac{\partial \theta}{\partial t} = \underbrace{\frac{\partial \theta_0}{\partial z_r}(z_r,t) \frac{\partial z_r}{\partial t}}_{I} + \underbrace{\frac{\partial \theta_0}{\partial t}(z_r,t)}_{II} + \underbrace{\frac{\partial \theta^{\prime}}{\partial t}({{{\mathbf{x}}}},t)}_{III} .$$

In this formulation, term (I) represents temperature changes due to vertical movements of *z*_*r*_ surfaces, which are primarily adiabatic in nature. Term (II) captures diabatic changes in the reference temperature profile *θ*_0_(*z*, *t*), while term (III) corresponds to spice, or density-compensated temperature changes. One way to ensure that *θ*_0_(*z*, *t*) and *S*_0_(*z*, *t*) depend on time only in the presence of diabatic sources or sinks of heat and salt is by defining them as thickness-weighted isopycnal averages^32^. In the absence of diabatic effects, term (II) would vanish, making term (I) unambiguously associated with adiabatic processes. We therefore refer to this term as ‘dynamic heave’. Term (II) arises from thickness-weighted isopycnally averaged diabatic temperature changes, which primarily affect density by modifying *ρ*_0_(*z*, *t*) without creating horizontal density gradients ${\rho }^{{\prime} }({\bf{x}},t)$ in Eq. (5). As such, it represents a dynamically inert form of heave, which we call ‘passive heave’.

Integrating terms (I) and (II) over time from specified initial conditions yields an explicit decomposition of temperature anomalies as:

9 $$\theta({{{\mathbf{x}}}},t) = \underbrace{\theta_{heave}^{dyn}({{{\mathbf{x}}}},t) + \theta_{heave}^{pas}({{{\mathbf{x}}}},t)}_{\theta_{heave}} + \theta^{\prime}({{{\mathbf{x}}}},t).$$

As detailed in Methods, we use monthly data, and therefore *θ*_0_(*z*_*r*_, *t*) is low-pass filtered in time so that the term (II) effectively retains the global warming trend by removing intra-seasonal to seasonal variations. We hypothesise that the passive heave component similarly corresponds to the ‘added heat’ identified in previous studies^10–12^. By construction, both the dynamic heave and spice components have negligible volume integrals in our framework, meaning that they represent redistribution mechanisms rather than net additions or removals of heat.

### Disentangling the mechanisms behind recent ocean warming

**Fig. 2:**
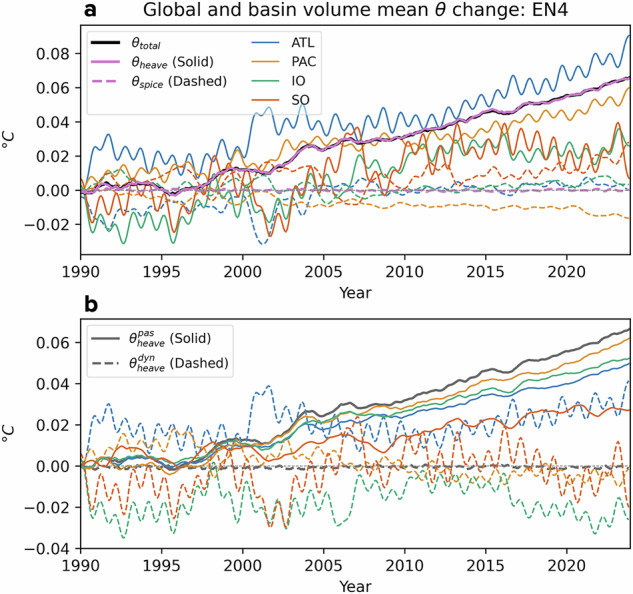
Global volume-weighted average of ocean potential temperature change during 1990–2024 relative to January 1990. **a** The total temperature change (*θ*_*total*_) is decomposed into heave (*θ*_*heave*_) and spice (*θ*_*spice*_) components, further partitioned among the four ocean basins: Atlantic, Pacific, Indian and Southern Oceans. The Southern Ocean is defined as south of 35°S. **b** The decomposition of total heave warming into passive (${\theta }_{heave}^{pas}$) and dynamic (${\theta }_{heave}^{dyn}$) components, also divided across the four ocean basins.

We used observational potential temperature and salinity from the EN4 dataset^33^ to compute the spice and heave contributions in our decomposition. This decomposition is further analysed across the four main ocean basins: the Atlantic, Pacific, Indian and Southern Oceans to explore basin-scale differences. Figure 2a shows the steady increase in globally volume-averaged ocean temperatures since the 1990s, interrupted by a temporary pause around the turn of the 21st century. From 2002 onward, the warming resumes nearly linearly at a rate of ~0.02 °C per decade. This global warming trend aligns with heave-driven warming, which reflects the net heat uptake associated with greenhouse gas emissions. In contrast, the volume-averaged spice component cancels out across the global ocean, resulting in no net contribution to the global mean temperature increase. Instead, it redistributes heat internally along isopycnals, creating spatial heterogeneity with small but distinct inter-basin differences. Across all basins, spice exhibits interannual variability without consistent decadal trends, except in the Pacific Ocean, where a net cooling trend is observed. Heave-driven warming, however, is persistent in the Atlantic and Pacific Oceans over the past 34 years, while the Southern and Indian Oceans show strong interannual-to-decadal oscillations that interrupt their underlying anthropogenic warming trends, particularly during 1990–2002. These fluctuations, potentially linked to climate modes or variability in ocean circulation, highlight the importance of disentangling long-term anthropogenic warming from short-term variability.

Figure 2b decomposes total heave into passive and dynamic contributions. The passive heave component captures the uptake of net excess heat, which increases the net mass of lighter density classes. This passive heave warming corresponds directly to the global ocean warming (Fig. 2b), serving as a diagnostic for detecting anthropogenic heat gain at both global and regional scales. Conversely, the dynamic heave component, which globally sums to zero, redistributes heat through adiabatic vertical displacements of *z*_*r*_ caused by factors such as wind stress perturbations, internal waves and ocean circulation changes. Unlike passive heave, dynamic heave neither adds nor removes heat from the system, operating independently of the long-term warming trend tied to greenhouse gas emissions.

On regional scales, significant interannual-to-decadal variability manifested in dynamic heave is superimposed on top of the sustained passive heave warming (Fig. 2b). Before 2005, dynamic heave dominated basin-scale temperature evolution, often exceeding passive heave warming in magnitude. However, since 2005, passive heave has become the primary driver of warming across all basins. The Pacific Ocean shows the fastest acceleration of passive heave warming, with negligible dynamic heave trends post-2005. The Southern Ocean, despite its known heat uptake potential, experiences only modest passive heat gain. While the Atlantic shows the most rapid total heave warming (Fig. 2a), it turns out that a substantial portion is attributed to dynamic heave with the isolated contribution from passive heave warming being comparatively smaller. In the Indian Ocean, much of the passive heave warming, which continues to absorb significant excess heat, is offset by dynamic heave cooling over the past two decades, resulting in only limited apparent warming as indicated by total heave warming. As global warming accelerates, passive heave is expected to increasingly dominate over dynamic heave. These findings, however, reveal how the complexities of natural variability and heat redistribution, represented by dynamic heave, can obscure the anthropogenic warming signal in recent observations.

**Fig. 3:**
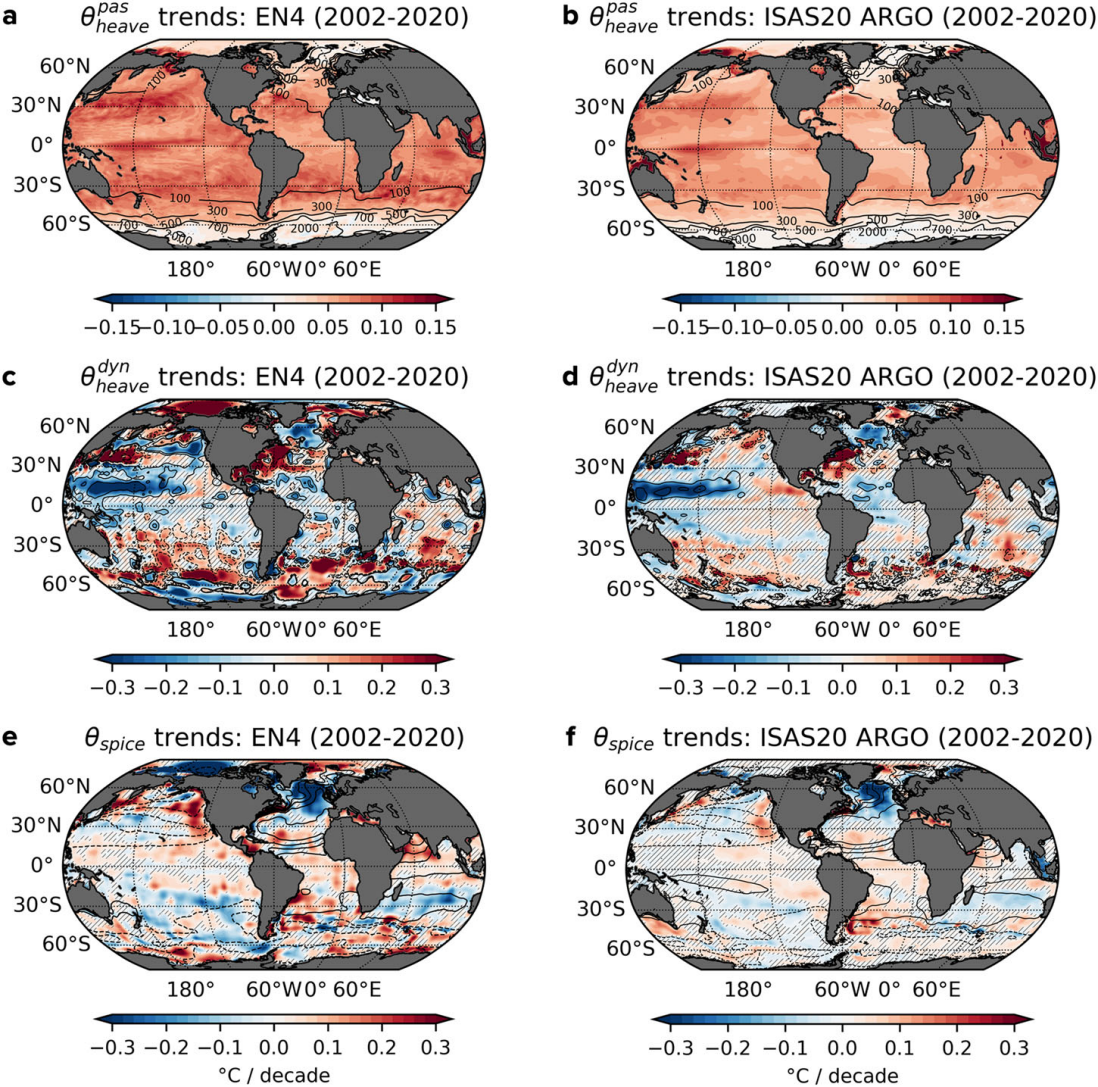
Maps of decomposed temperature trends in the upper 700 m (2002–2020). **a**, **b** Temperature trends averaged over the upper 700 m in the EN4 and ISAS20 ARGO datasets, attributed to the passive heave component, ${\theta }_{heave}^{pas}$. Contours indicate the outcropping time-mean reference depth *z*_*r*_ (in meters). **c**, **d** As in (**a**, **b**), but for the dynamic heave component, ${\theta }_{heave}^{dyn}$. Contours indicate *z*_*r*_ trends of ±5 and ±10 m per decade, averaged over 0–700 m depth range. **e**, **f** As in (**a**–**d**), but for the spice component, *θ*_*spice*_, with contours marking climatological spice levels ranging from −5 °C to 5 °C in 1 °C intervals. Hatching shows regions where trends are not statistically significant at *p* ≤ 0.05.

To assess the sensitivity of our decomposition to the choice of observational products, we evaluated the three components, ${\theta }_{heave}^{pas}$, ${\theta }_{heave}^{dyn}$ and *θ*_*spice*_, using both the EN4 and ISAS20 ARGO datasets^33,34^. Figure 3 presents the spatial patterns of temperature trends for each component in the upper 700 m during the period 2002-2020. The ${\theta }_{heave}^{pas}$ trends manifest as large-scale, basin-wide warming, particularly pronounced in the equatorial and midlatitude regions (Fig. 3a, b). This contrasts with localised, mesoscale variability observed in ${\theta }_{heave}^{dyn}$ trends (Fig. 3c, d), likely driven by eddy dynamics and internal waves^35^.

**Fig. 4:**
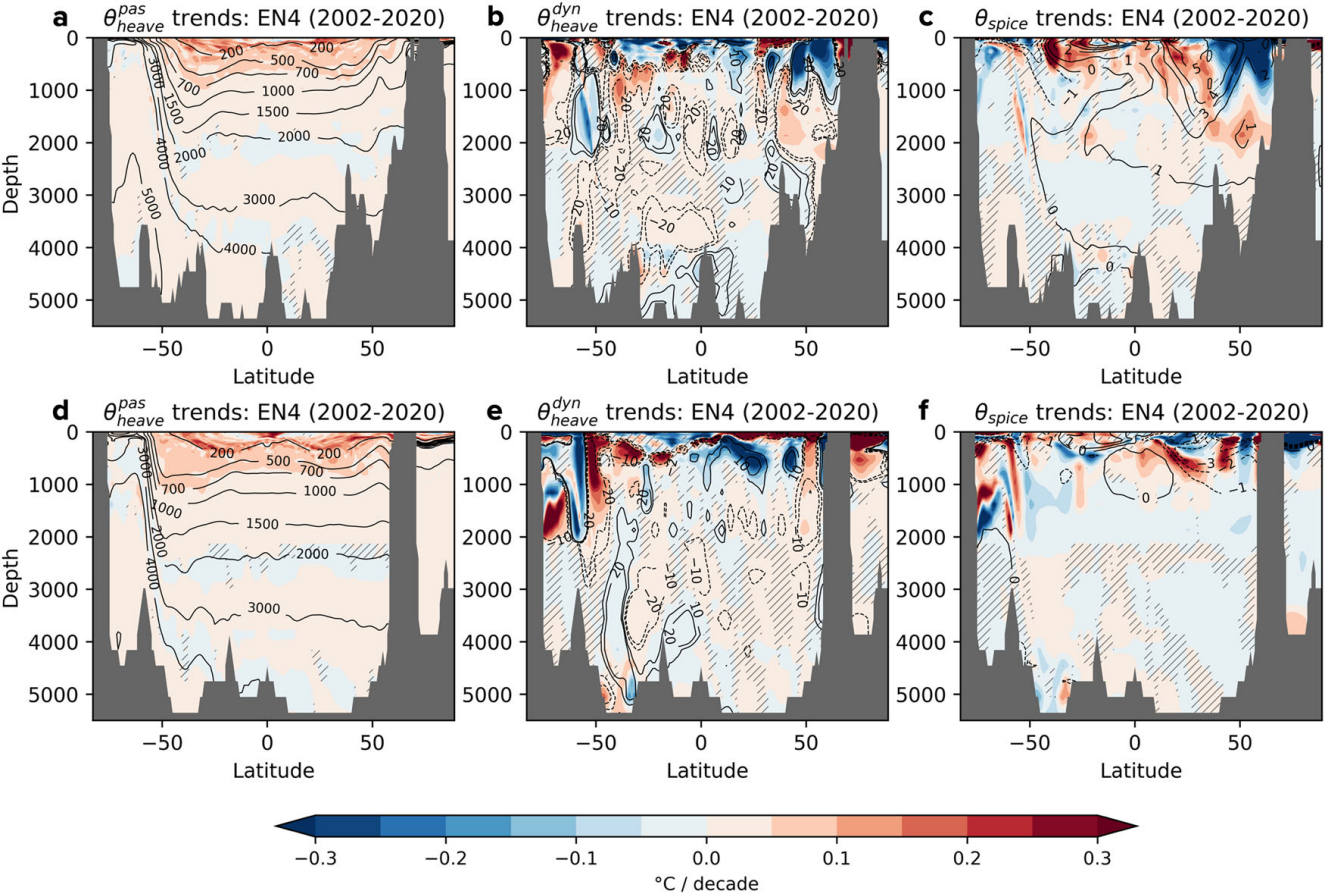
Decomposed temperature trends along meridional sections at 30°W and 210°E (2002–2020). **a** EN4 temperature trends attributed to the passive heave component, ${\theta }_{heave}^{pas}$, along 30°W in the Atlantic, with contours representing the mean reference depth, *z*_*r*_ (in meters) along this section. **b** As in (**a**), but showing trends due to dynamic heave, ${\theta }_{heave}^{dyn}$, with contours indicating *z*_*r*_ trends at intervals of ±10 m and ±20 m per decade. **c** As in (**a**, **b**), but for the spice component, *θ*_*spice*_, with contours indicating the climatological spice values in °C. **d**–**f** As in (**a**–**c**), but for a meridional section at 210°E in the Pacific. Hatching marks regions where trends are not statistically significant at *p* ≤ 0.05.

On interannual to decadal timescales, passive heave warming is noticeably absent or limited south of the Antarctic Circumpolar Current (ACC) and in the subpolar North Atlantic, whereas it intensifies in subtropical gyres and the equatorial Pacific (Fig. 3a, b). These spatial pattern have persisted over the past 50 years in the EN4 dataset (Supplementary Fig. 1). Both datasets exhibit consistent large-scale patterns of passive heave warming; however, EN4 shows significantly stronger warming than ISAS20 ARGO, with global trends of 0.064 °C/decade in EN4 compared to 0.044 °C/decade in ISAS20 ARGO in the upper 700 m. The muted warming poleward of *z*_*r*_ = 700 m outcrops in the Southern Ocean and North Atlantic suggests that surface warm anomalies are subducted along outcropping isopycnals and subsequently advected into the subtropical interior thermocline, where passive heave warming intensifies. This process is especially evident in the meridional plane of passive heave warming trends (Fig. 4a, d), which reveal subtropical gyre signals driven by the subduction of surface warming into the subtropical interior via Ekman pumping. Corroborating this, enhanced ${\theta }_{heave}^{pas}$ trends within the subtropical gyres align with patterns of mean wind stress curl and the associated climatological downwelling (see Supplementary Fig. 2). Heat subducted and transported via the subsurface branches of the subtropical cells ultimately contributes to the warming of the equatorial thermocline.

Interestingly, despite high-latitude regions sharing similar outcropping features, passive heave warming shows a striking asymmetry: it is markedly suppressed south of the ACC, while northern high latitudes exhibit subtle warming signals penetrating into the interior over the 19-year period (Fig. 4a, d). This asymmetry suggests that anomalous surface warming south of the ACC is predominantly exported equatorward through the upper branch of the Southern Ocean’s meridional overturning circulation before being subducted into the interior^36,37^. In the northern high-latitudes, the subpolar North Pacific experiences stronger warming than the subpolar North Atlantic (Figs. 3a, b and 4a, d). This difference may be attributed to the outcropping of iso-*z*_*r*_ surfaces in the Atlantic but not in the Pacific, allowing for heat subduction along tilted isopycnals in the Atlantic, whereas warming in the Pacific tends to remain more horizontally confined along flatter isopycnals. Furthermore, Fig. 3a, b reveals intensified near-surface warming in the equatorial Pacific, largely restricted to the upper 100 m (Fig. 4d). Taken together, these features highlight how anthropogenic diabatic warming signals are distinctly captured by the passive heave component, which is inherently different from heat redistribution mechanisms.

The dynamic heave component captures the redistribution aspect of the total heave signal, primarily associated with adiabatic undulations of iso-*z*_*r*_ surfaces. Variations in mechanical wind forcing can significantly deform isopycnals, adjusting iso-*z*_*r*_ surfaces such that their deepening (shoaling) corresponds to adiabatic warming (cooling) (Fig. 3c, d). Statistically significant trends in dynamic heave are broadly consistent between the EN4 and ISAS20 datasets, though polar regions exhibit greater divergence due to a paucity of observations. Here, we focus on the common features to highlight robust dynamical responses, which exhibit enhanced *z*_*r*_ changes and dynamic heave signals in regions with a stronger influence of ocean dynamics. Dynamic heave warming between 40°S and 60°S is linked to the deepening of *z*_*r*_ surfaces, or negative *z*_*r*_ trends, flanked by subsurface cooling zones to the south where *z*_*r*_ shoals (Fig. 3c, d). These patterns closely mirror trends in Ekman pumping, which show increased wind-driven downwelling near the ACC and upwelling poleward (see Supplementary Fig. 2)^38,39^. This adiabatic redistribution is the dominant driver of Southern Ocean warming among the three decomposition components, contributing to significant regional and interannual variability yet having no net impact on long-term basin-wide warming (Fig. 2b). In the North Atlantic, a distinct tripole pattern is evident, marked by cooling due to shoaling isopycnals within the subpolar region, warming from sinking isopycnals near the Gulf Stream and cooling again from rising isopycnals in the tropical Atlantic. This configuration resembles the positive phase of NAO^40–42^. While a detailed analysis of the NAO is beyond the scope of this study, observed wind changes partially validate *z*_*r*_ trends: intensified positive wind stress curl in the tropics and the northeast subpolar gyre promotes Ekman upwelling, whereas stronger negative wind stress curl in the subtropical North Atlantic enhances downwelling (Supplementary Fig. 2). A comparable north-south dipole also appears in the North Pacific, where cooling dominates subtropical cell upwelling regions, while warming is seen near subtropical mode water, potentially driven by the northward migration of wind stress around the Kuroshio Extension^43^ (Fig. 3c, d).

Density-compensated spice anomalies are expected to act as passive tracers outside polar oceans depending on the neutrality of the Lorenz reference density surfaces^44^. Climatological spice patterns reflect known ocean water mass distributions, as shown by the contour lines in Fig. 4c, f. For instance, along the Atlantic at 30°W, positive *θ*_*spice*_ denotes warm, salty waters like North Atlantic Deep Water and Mediterranean Water, while negative *θ*_*spice*_ represents cooler, fresher waters such as Antarctic Intermediate Water and Antarctic Bottom Water (Fig. 4c). Consequently, *θ*_*spice*_ trends directly capture changes in water mass properties. Over the recent 19-year period, significant spice temperature trends are concentrated in the upper 2000 m, with deep water masses like North Atlantic Deep Water and Antarctic Bottom Water showing minimal spice trends. Notably, strong cooling of *θ*_*spice*_, indicative of freshening, occurs in the upper 1000 m of the northern North Atlantic and Antarctic Intermediate Water (Fig. 4c). Conversely, warming and salinification dominate at lower latitudes in both hemispheres. In the subpolar North Atlantic, dynamic heave and spice components combine to reinforce cooling by redistributing heat southward, as seen in both the EN4 and ISAS20 datasets (Fig. 3c–f); however, only dynamic heave directly influences ocean dynamics by creating density gradients. The water mass properties in the subpolar Atlantic seem to originate from the subtropics, as evidenced by the climatological warm spice in Fig. 3e, f, where warm, salty waters peak in the subtropics and are transported by the Atlantic Meridional Overturning Circulation (AMOC) into the northeastern subpolar Atlantic. Therefore, subpolar cooling (freshening) likely reflects a reduced northward transport of warm, saline waters through the AMOC’s upper branch. A weakening AMOC may also explain the pronounced warming and salinification confined to the upper western boundary of the North Atlantic (Fig. 3e, f)^45^.

### Climate model representation of ocean warming mechanisms

Heave and spice, as defined in this study, relate to distinct physical processes that may not be equally well captured in observational products. Indeed, the EN4 and ISAS datasets analysed here rely on statistical interpolation strategies, which lack explicit dynamical constraints, particularly in regions with sparse observational coverage. As a consequence, the dynamic heave component, which directly reflects ocean dynamic processes like wind-driven waves and eddies, is particularly susceptible to observational uncertainties. To better demonstrate the robustness of the decomposition, it is essential to examine heave and spice components derived from dynamically consistent numerical models. To this end, we apply the temperature decomposition to historical simulations from two state-of-the-art CMIP6 models, GFDL-CM4 and NCAR-CESM2, to identify potential areas for improvement.

**Fig. 5:**
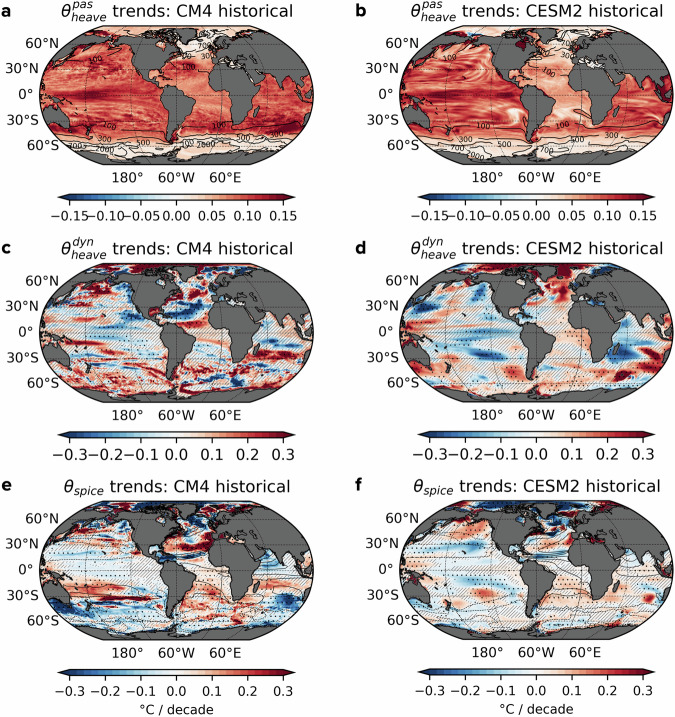
Maps of decomposed temperature trends in the upper 700 m from climate simulations. **a**, **b** Temperature trends attributed to the passive heave component, averaged over the 0–700 m, derived from CMIP6 historical experiments (2000–2014) using GFDL-CM4 and NCAR-CESM2 models. Contours represent the outcropping time-mean reference depth *z*_*r*_ (in meters). **c**, **d** As in (**a**, **b**), but for the dynamic heave component. **e**, **f** As in (**a**–**d**), but for the spice component, with contours indicating climatological spice levels ranging from −5 °C to 5 °C in 1 °C intervals. Stippling in (**c**–**f**) highlights areas where temperature trends share the same sign as those in the EN4 dataset over the same period. Hatching marks regions where trends are not statistically significant at *p* ≤ 0.05.

Both CM4 and CESM2 successfully reproduce key features of passive heave warming observed in EN4, including the limited warming outside the *z*_*r*_ = 700 m outcrops in the Southern Ocean and northern North Atlantic, as well as the amplified warming within subtropical gyres and the western equatorial Pacific (Fig. 5a, b). However, both models appear to overestimate the magnitude of passive heave warming in the upper 700 m of key regions, with global trends of 0.078 °C/decade in CM4 and 0.072 °C/decade in CESM2, exceeding the observed trend of 0.059 °C/decade in EN4. Notably, CESM2 displays weaker passive heave warming than CM4 in the Atlantic subtropical gyres. Under increased greenhouse gas forcing, as demonstrated in the 1pctCO_2_ experiment and SSP scenarios, passive heave warming emerges as the dominant contributor to ocean heat uptake, both in terms of magnitude and spatial patterns, compared to other redistribution processes (see Supplementary Fig. 3). These spatial patterns, such as the suppressed warming in the high-latitude Southern Ocean and subpolar North Atlantic, remain consistent with those during the warming phase of historical simulations and observations, reinforcing the validity of passive heave warming signals as a response to greenhouse gas forcing.

Both models reasonably replicate climatological spice distributions, as indicated by the solid black contour lines in Fig. 5e, f compared to Fig. 3e, f. However, they struggle to fully capture observed *θ*_*spice*_ trends, particularly outside the Atlantic. In the Atlantic, there is reasonable agreement between models and observations, capturing cooling and freshening in the subpolar region, warming and salinification along the western boundary and broad warming in the subtropical Atlantic. While regional discrepancies in spice trends can contribute to model biases and spread in simulated regional ocean warming, their impact on ocean dynamics is limited, as spice primarily acts as a passive tracer of water mass properties. In contrast, the dynamic heave component provides a more dynamic perspective on ocean warming mechanisms. Trends in ${\theta }_{heave}^{dyn}$ show partial agreement between models and observations (stippling in Fig. 5c, d), with expected mismatches arising in part from the models’ inability to fully replicate observed climate variability due to chaotic internal dynamics and uncertainties in external forcing agents^46–48^. One of the key roles of dynamic heave is its contribution to the Southern Ocean warming, a feature that both models capture reasonably. However, CESM2, with its coarse resolution and lack of explicit mesoscale eddy representation, underestimates the spatial variability spanning the Southern Ocean. In the Pacific, dynamic heave patterns in both models resemble the cool phase of ENSO, but inter-model disagreements persist in other regions despite identical historical forcing, reflecting the inherent variability of unforced dynamics within each model.

### Implications for global and regional sea-level change

Our decomposition of *θ*_*heave*_ into passive and dynamic components has important implications for elucidating the relative contributions of anthropogenic heat uptake and heat redistribution in shaping global and regional sea-level changes. The passive heave component, by construction, represents the temperature signals that govern the evolution of the mean density profile through air-sea interactions at density outcrops, followed by vertical mixing and heat advection. Consequently, passive heave warming increases the thickness of lighter density layers, which directly contribute to global mean sea-level rise via thermal expansion. In contrast, the dynamic heave component captures the temperature changes resulting from local mass or volume reorganisation, associated with predominantly adiabatic vertical movements of the iso-*z*_*r*_ surfaces. These redistributions are primarily driven by dynamical responses, including changes in wind stress, mesoscale eddies and wave-driven processes such as Rossby and internal waves. As a result, dynamic heave is associated with regional sea-level changes and variability without contributing to global mean changes. In numerical ocean circulation models, sea-level variations are typically represented as the sum of dynamic sea level (DSL, variable ‘zos’ in CMIP terminology) and a global mean correction, which is diagnosed a posteriori to satisfy mass conservation. DSL, which globally averages to zero by definition, is closely linked to dynamic heave, while the global mean correction reflects the contribution of passive heave warming.

**Fig. 6:**
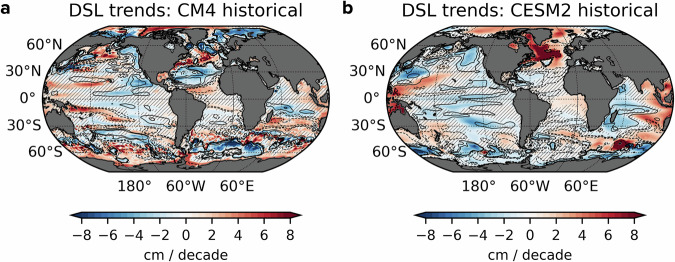
Trends in ocean dynamic sea level (DSL) from climate simulations. DSL trends, defined as the difference between local sea surface height relative to the geoid and its global ocean mean, are derived from CMIP6 historical experiments (2000–2014) using (**a**) GFDL-CM4 and **b** NCAR-CESM2 models. Contours indicate *z*_*r*_ trends of ±2, ±4, ±8 and ±50 m per decade per decade, averaged over 0–700 m depth range. Hatching indicates regions where trends are not statistically significant at *p* ≤ 0.05.

Unlike passive heave and spice components, which predominantly act as passive tracers of water mass changes, dynamic heave actively alters the ocean’s density structure, i.e. ${\rho }^{{\prime} }({\bf{x}},t)$ in Eq. (5), which modulates the baroclinic pressure gradients and, in turn, geostrophic flows. This dynamical linkage ties dynamic heave to regional DSL variability and redistribution effects. As illustrated in Fig. 6, DSL trends from CM4 and CESM2 reveal substantial inter-model differences in spatial patterns, a persistent issue in modelling that contributes to significant uncertainties in sea-level projections^49,50^. Regions with downward (negative) trends in *z*_*r*_ experience expansion of the upper ocean layers, leading to dynamic heave warming and elevated DSL, whereas upward (positive) *z*_*r*_ trends correspond to cooling and decreased DSL. The intrinsic relationship between DSL and dynamic heave temperature trends is quantified by strong pattern correlations; *R* ≈ 0.70 in CM4 and *R* ≈ 0.81 in CESM2. This remarkable correspondence substantiates the hypothesis that dynamic heave component uniquely captures the dynamical aspects of ocean warming tied to changes in horizontal density gradients and ocean circulation. Therefore, reliable projections of regional sea-level changes under anthropogenic climate change critically hinge on improving simulations and understanding of the dynamic heave component.

## Discussion

Our physically based decomposition of temperature variability unravels the mechanisms underlying recent ocean warming, partitioning temperature changes into three distinct components: density-compensated ‘spice’ and density-contributing ‘heave’, with heave further divided into ‘passive’ and ‘dynamic’ contributions. This approach provides unprecedented clarity in understanding anthropogenic heat uptake and redistribution, complementing model experiments that use perturbative surface temperature anomalies based on climatological circulation, which may fall short of replicating real-world oceanic processes. The robustness of our framework lies in its foundation on Lorenz reference density *ρ*_0_(*z*_*r*_, *t*) and reference depths *z*_*r*_(*S*, *θ*, *t*), which objectively define density surfaces and establish a fundamental link to the passive and dynamic components of temperature changes. This method, grounded in dynamical principles, ensures a physically consistent analysis of ocean heat uptake across both observations and climate models.

The passive heave warming, directly driven by heat added via anthropogenic air-sea heat flux increases, accounts for the entirety of global ocean warming. Its spatial distribution reveals the subduction of heat along iso-*z*_*r*_ surfaces as well as its diffusion across isopycnals, accompanied by modifications in the reference profile *θ*_0_(*z*, *t*). This dynamically passive process does not involve the actual movement of fluid parcels relative to their reference positions *z*_*r*_, but evolves as a distinct signature of anthropogenic heat gain. Despite a fundamental difference in methodology, our findings broadly align with earlier studies on passive ocean heat uptake^10–12,14,18^. Even though previous studies show some variation in estimates of passive warming, they consistently identify subtropical warming peaks driven by surface Ekman convergence—a feature corroborated by our results. Our analysis extends these findings by further characterising key regional patterns of passive heave, including minimal warming south of the ACC and in the subpolar North Atlantic as well as intensified equatorial Pacific warming near the surface, likely resulting from the vertical mixing of warmer surface waters.

The dynamic heave component represents heat redistribution through changes in dynamical processes, which consequently alter density gradients and ocean circulations. Despite its strong spatial and interannual-to-decadal variability, a warming signal in the Southern Ocean emerges as a persistent feature of dynamic heave, evident in both observations and historical climate simulations, and projected to continue under future warming scenarios. Note that dynamic heave may be partially affected by small diabatic effects linked to variable mixing processes and north-south seasonal asymmetries, potentially arising from either anthropogenic or natural sources. However, these diabatic contributions are likely minimal in our analysis of interannual-to-decadal variability and multi-decadal trends. Accurately observing or simulating dynamic heave remains a difficult yet critical task due to its reliance on mesoscale eddies, internal wave dynamics and chaotic internal variability, which are rarely, if ever, satisfactorily resolved in models and observations.

The spice component of temperature variability analysed in this study appears comparable to that discussed in previous studies^9,23,40^, despite differences in the covered periods or the specific definitions of spice. We use a fundamental definition of spice that does not require orthogonality to density^44^; however, whether spice should be generically defined as a redistribution mechanism remains an open question, particularly given the lack of consensus on its optimal definition. By definition, spice exerts no influence on density or ocean dynamics, primarily behaving as a passive tracer. Nevertheless, it may not be exactly passive in the polar oceans and Gulf Stream, where the Lorenz reference density departs from neutrality due to the thermobaric nonlinearity of the equation of state. Outside these regions, the spice component does not affect in-situ density, making its potential impact on sea-level changes negligible.

Our study sheds light on how the ocean stores and redistributes heat in response to increased greenhouse gas emissions, highlighting its implications for sea-level change. The heave component, strongly linked to thermal expansion, stands out as a major driver of global sea-level rise. By distinguishing between passive and dynamic contributions, we can better differentiate regions where sea-level rise originates from anthropogenic heat uptake versus those influenced by dynamical changes. Looking forward, though historically less influential than redistribution effects at regional scales, the passive heave signal is expected to intensify and become a dominant factor as ocean warming continues (Supplementary Fig. 3)^15^. Nonetheless, it is equally important to acknowledge the central role of dynamic heave in shaping localised climate impacts, such as ocean circulation changes and regional sea-level variability. By providing a comprehensive, physically based framework, our study aims to advance the ability to predict and mitigate the impacts of climate change on both global and regional scales.

## Methods

### Lorenz reference state of the ocean

The Lorenz reference state provides a theoretical construct that simplifies the analysis of ocean dynamics by establishing a baseline of minimum potential energy through adiabatic rearrangement of fluid parcels. This stably stratified state has thus zero horizontal density gradients, making it a useful reference for understanding the distribution and movement of water masses. In this reference state, the density, *ρ*_0_(*z*, *t*), is a one-dimensional function of depth *z* that evolves over time *t*, reflecting changes in overall stratification of the ocean. Physically, the Lorenz reference density profile *ρ*_0_(*z*, *t*) is obtained by means of an adiabatic and iso-haline re-arrangement of mass (or volume, in the Boussinesq approximation). For a realistic nonlinear equation of state, this can be efficiently achieved by first computing the probability density function *Π*(*S*, *θ*) defining thermohaline volume in predetermined (*S*, *θ*) bins, and mapping volume in (*S*, *θ*) space into physical space at predetermined target depths, as discussed by ref. ^27^.

Once the reference density profile is established, the reference depth *z*_*r*_(**x**) for each ocean parcel can be determined using the level of neutral buoyancy equation^29^:

10 $$\rho [S,\theta ,{p}_{0}({z}_{r},t)]={\rho }_{0}({z}_{r},t),$$

where *p*_0_(*z*, *t*) is such that *gρ*_0_(*z*, *t*) = −*dp*_0_/*dz*(*z*, *t*). This defines *z*_*r*_(*S*, *θ*, *t*) at which an ocean parcel, brought adiabatically from its original position, matches the reference density *ρ*_0_(*z*_*r*_, *t*). Thus, *z*_*r*_ represents the notional position where the parcel would be in a state of rest (e.g. in equilibrium) within the Lorenz reference stratification, confined between the ocean surface and bottom.

### Defining spice and heave anomalies

By construction, parcels at the same reference depth *z*_*r*_ share the same reference density, leading to the relationship:

11 $$\rho (S,\theta ,{p}_{0}({z}_{r},t))=\rho ({S}_{0}({z}_{r},t),{\theta }_{0}({z}_{r},t),{p}_{0}({z}_{r},t)),$$

where *S*_0_ and *θ*_0_ denotes the thickness-weighted isopycnal average salinity and temperature, respectively along surfaces of constant reference density. This equation indicates that, at the reference pressure *p*_*r*_ = *p*_0_(*z*_*r*_, *t*), a fluid parcel with *S* and *θ* has the same density as a parcel with *θ*_0_(*z*_*r*_, *t*) and *S*_0_(*z*_*r*_, *t*). Thus, *θ*_0_(*z*_*r*_, *t*) and *S*_0_(*z*_*r*_, *t*) represent the components of *θ* and *S* that in tandem determine density for any given time t. This density-contributing component, or ‘heave’ describes temperature and salinity changes associated with the vertical displacement of isopycnals, capturing alterations in the ocean’s density structure. The deviations, ${\theta }^{{\prime} }({\bf{x}},t)=\theta ({\bf{x}},t)-{\theta }_{0}({z}_{r}({\bf{x}}),t)$ and ${S}^{{\prime} }({\bf{x}},t)=S({\bf{x}},t)-{S}_{0}({z}_{r}({\bf{x}}),t)$, are density-compensated anomalies, or ‘spice’. They represent variations along the *S* − *θ* curve that do not directly affect density, approximately satisfying the relationship:

12 $${\alpha }_{\gamma }{\theta }^{{\prime} }\approx {\beta }_{\gamma }{S}^{{\prime} },$$

where *α*_*γ*_ = *α*(*S*_0_(*z*_*r*_, *t*), *θ*_0_(*z*_*r*_, *t*), *p*_0_(*z*_*r*_, *t*)) and *β*_*γ*_ = *β*(*S*_0_(*z*_*r*_, *t*), *θ*_0_(*z*_*r*_, *t*), *p*_0_(*z*_*r*_, *t*)) are the thermal expansion and haline contraction coefficients, respectively. Therefore, the temperature field *θ* can be decomposed into two components: *θ*_*heave*_ = *θ*_0_(*z*_*r*_, *t*) and *θ*_*spice*_ = *θ* − *θ*_0_(*z*_*r*_, *t*). Note that the heave component should be evaluated relative to a reference time *t*_0_, expressed as *θ*_0_(*z*_*r*_(**x**), *t*) − *θ*_0_(*z*_*r*_(**x**), *t*_0_) because, by definition, heave represents the temperature change linked to the vertical displacement of its reference depth (i.e. reference density).

### Heave anomalies due to diabatic and adiabatic processes

As shown, heave anomalies encapsulates both changes due to the shift in *z*_*r*_ of each parcel and temporal variations in *θ*_0_(*z*, *t*). To distinguish between these two effects, we take the time derivative of the temperature field decomposition: *θ*(**x**, *t*) = *θ*_0_(*z*_*r*_, *t*) + *θ*_*spice*_(**x**, *t*). Using the chain rule, we can express the time evolution of temperature as:

13 $$\frac{\partial \theta }{\partial t}=\frac{\partial {\theta }_{0}}{\partial t}({z}_{r},t)+\frac{\partial {\theta }_{0}}{\partial {z}_{r}}({z}_{r},t)\frac{\partial {z}_{r}}{\partial t}+\frac{\partial {\theta }_{spice}}{\partial t}.$$

This equation separates the heave component into diabatic and adiabatic terms, detailed as follows:

- The term $\frac{\partial {\theta }_{0}}{\partial t}({z}_{r},t)$ represents the primarily diabatic passive heaving mode. It describes the rate of change of temperature at a fixed *z*_*r*_ with time due to the reference temperature profile change *θ*_0_(*z*, *t*), which is only modulated by external heating or cooling, as well as interior irreversible mixing processes. This term captures changes in the average temperature of the water mass occupying a given *z*_*r*_. Since the reference state is constructed to remove effects of adiabatic processes, any change in *θ*_0_(*z*, *t*) at a constant *z* must be due to diabatic warming or cooling.
- The term $\frac{\partial {\theta }_{0}}{\partial {z}_{r}}({z}_{r},t)\frac{\partial {z}_{r}}{\partial t}$ captures the primarily adiabatic dynamic heave response. It reflects how changes in *z*_*r*_ due to the physical movement of water parcels, without any heat exchange with the environment, translates into temperature changes based on the reference temperature gradient of *θ*_0_(*z*, *t*). Note that any purely adiabatic changes to *z*_*r*_ would result in no net change in the reference stratification *θ*_0_(*z*, *t*) that results from adiabatic rearrangement. Adiabatic vertical displacements of *z*_*r*_ are not associated with any real change in water mass properties, but rather occur through the reorganisation of water masses by changes in ocean dynamics.
- Finally, the term $\frac{\partial {\theta }_{spice}}{\partial t}$ represents density-compensated temperature variations.

In practice, the passive component in Eq. (13) is calculated as:

14 $$\begin{array}{ll}{\left(\frac{\partial {\theta }_{0}}{\partial t}\right)}^{n+1/2}({z}_{r},t)\,=\,\frac{1}{\Delta t}\left({\theta }_{0}[{z}_{r}({\bf{x}},t),t+\Delta t]-{\theta }_{0}[{z}_{r}({\bf{x}},t),t]\right)\\\qquad\qquad\qquad\qquad\approx \,{\left(\frac{\partial }{\partial t}\Delta {\theta }_{heave}^{pas}\right)}^{n+1/2},\end{array}$$

where *n* = 0, 1, . . . , *t* − 1. We thus obtain the passive and dynamic components of temperature change:

15 $$\Delta {\theta }_{heave}^{pas}[{\bf{x}},\,n+1]=\Delta {\theta }_{heave}^{pas}[{\bf{x}},\,n]+{\left(\frac{\partial {\theta }_{0}}{\partial t}\right)}^{n+1/2}({z}_{r},t)\,\Delta t$$

16 $$\Delta {\theta }_{heave}^{dyn}[{\bf{x}},\,n]={\theta }_{0}[{z}_{r}({\bf{x}},n),\,n]-\Delta {\theta }_{heave}^{pas}[{\bf{x}},\,n]$$

The calculations in Eqs. (15) and (16) require specifying initial conditions for the dynamic and passive heave components. For example, we set: $\Delta {\theta }_{heave}^{pas}[{\bf{x}},n=0]=0$ and thus $\Delta {\theta }_{heave}^{dyn}[{\bf{x}},n=0]={\theta }_{0}[{z}_{r}({\bf{x}},n=0),n=0]$, representing the total heave at the initial time step. Alternatively, the initial conditions can be reversed without affecting the results. This is because the heave definition is based on isopycnal displacements relative to a reference time, causing any initial condition effects to cancel out by construction. As a result, trends and anomalies in each component remain independent of the specific initial condition choice.

In principle, the reference temperature and salinity profiles in monthly timescale, *θ*_0_(*z*, *t*) and *S*_0_(*z*, *t*), experience intra-seasonal to seasonal fluctuations due to changes in solar radiation, precipitation and evaporation or short-term variations in mixing and advection. These transient variations are undesirable when aiming to isolate long-term global warming signals. To reduce short-term fluctuations, we apply a 12-month smoothing filter to the time series of *θ*_0_(*z*, *t*), allowing for a clearer focus on the sustained warming trends associated with anthropogenic climate change.

### Observational and model data

Observational estimates of temperature and salinity are obtained from the Enact Ensemble V.4.2.2 (hereafter EN4) and the In Situ Analysis System 20 (ISAS20 ARGO). EN4 provides data on a 1° × 1° horizontal resolution with 42 vertical levels^33^, while ISAS20 ARGO offers a finer 0.5° × 0.5° horizontal grid with 187 depth^34^. The EN4 dataset is constructed using data from Argo, ASBO (Arctic Synoptic Basinwide Oceanography), GTSPP (Global Temperature and Salinity Profile Program) and WOD13 (World Ocean Database). The ISAS20 gridded fields are solely based on Argo and Deep-Argo temperature and salinity observations, covering the period from 2002-2020. For consistency, we analyse monthly data from both EN4 and ISAS20 over their common maximised period 2002-2020.

For model-based analysis, we use outputs from historical simulations (2000–2014) conducted with the GFDL-CM4 and NCAR-CESM2 models, both part of phase 6 of the Coupled Model Intercomparison Project (CMIP6). The ocean component of GFDL-CM4 employs a nominal 0.25° horizontal grid spacing with 75 vertical levels. This eddy-permitting configuration of CM4 omits mesoscale eddy parameterisation. In contrast, CESM2 features a nominal 1° horizontal resolution with 60 vertical levels and includes advective and diffusive mesoscale eddy parameterisations. For our analysis, the native resolution outputs of both models are regridded onto a regular 1° longitude-latitude grid. To support the main results from the historical runs, we also analyse additional CMIP6 simulations, including piControl, 1pctCO_2_ and SSP5-8.5 scenario runs (see Supplementary Fig. 3).

## Supplementary information

Supplementary information

## Data Availability

The EN4.2.2 and ISAS20 ARGO data are publicly available at https://www.metoffice.gov.uk/hadobs/en4/download-en4-2-2.html and 10.17882/52367, respectively. The CMIP6 simulations from the CM4 and CESM2 models can be accessed at https://esgf-node.llnl.gov/projects/cmip6/.

## Code availability

The source code for the decomposition method is available at 10.5281/zenodo.14568523.
