# Supplementary material for: Disentangling anthropogenic and dynamic contributions to recent ocean warming
Source: NPJ Clim Atmos Sci. 2025 Apr 26;8(1):157. doi: 10.1038/s41612-025-01043-7 (PMC12033083; doi:10.1038/s41612-025-01043-7)
Supplement: Supplementary file 1 — Supplementary information [file 41612_2025_1043_MOESM1_ESM.pdf]

npj | climate and atmospheric science

Supplementary Information for "Disentangling anthropogenic and dynamic contributions to recent ocean warming"

Jiheun Lee[1,2], Rémi Tailleux[1], Till Kuhlbrodt[1,2],

[1]Department of Meteorology, University of Reading, Reading, UK

[2]National Centre for Atmospheric Science, UK

Contents of this file

1. Supplementary Figures 1 to 3

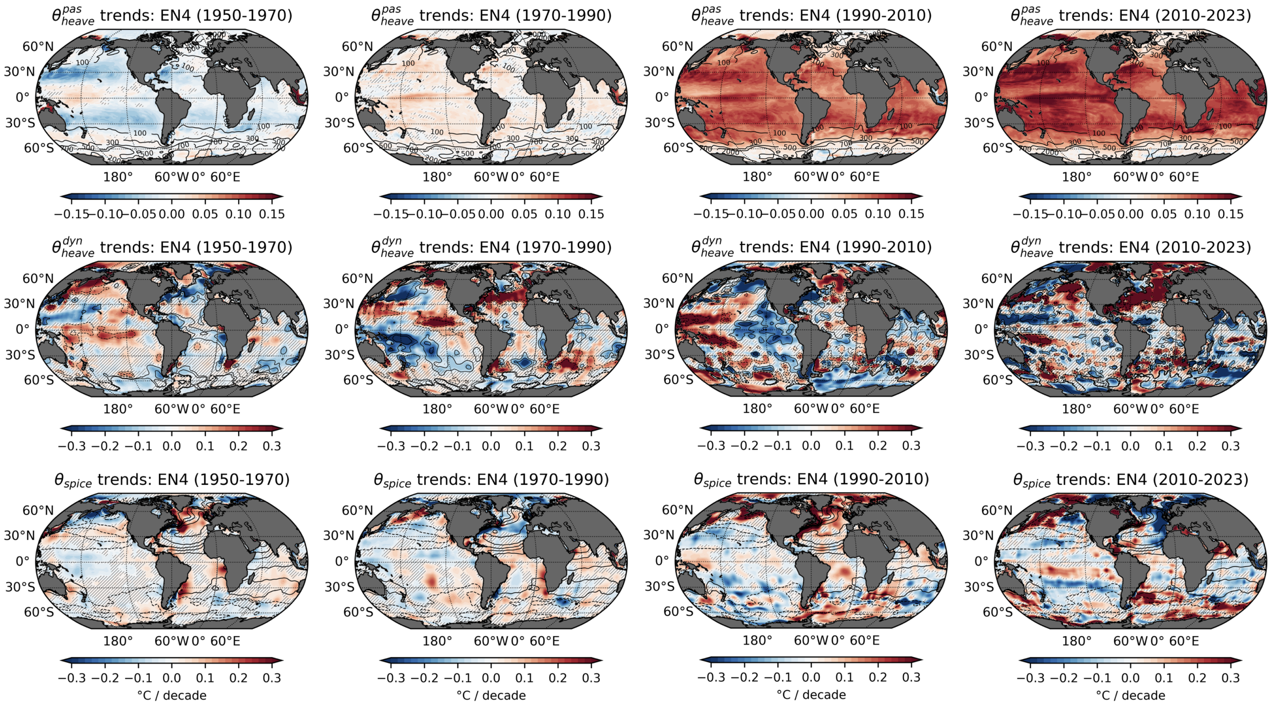

Supplementary Figure 1. Same as Figure 2 in the main text, but showing results for the EN4 dataset across four distinct periods: 1950-1970, 1970-1990, 1990-2010 and 2010-2023.

.

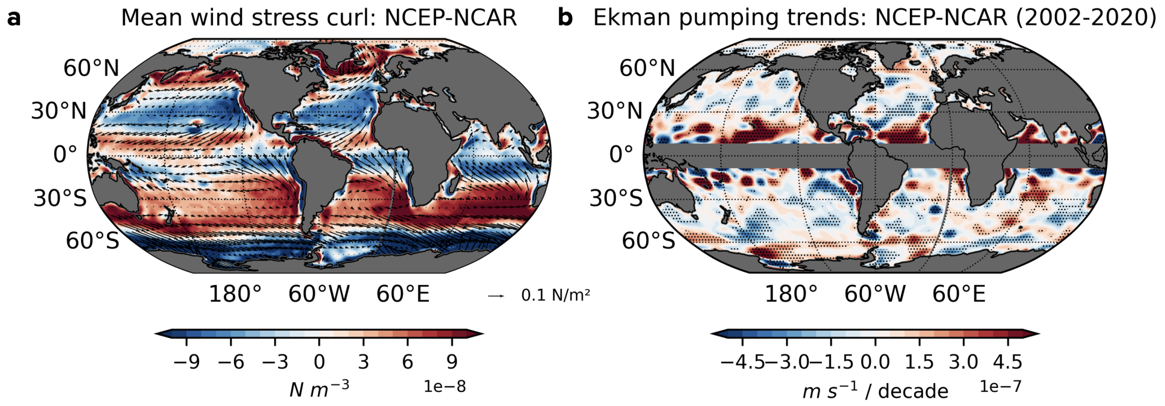

Supplementary Figure 2. **a,** Mean stress curl field and corresponding mean wind stress vector field derived from the NCEP-NCAR Reanalysis, covering the period 2002-2020. **b,** Trend in Ekman pumping, with stippling indicating regions where trends are statistically significant at $p \leq 0.05$.

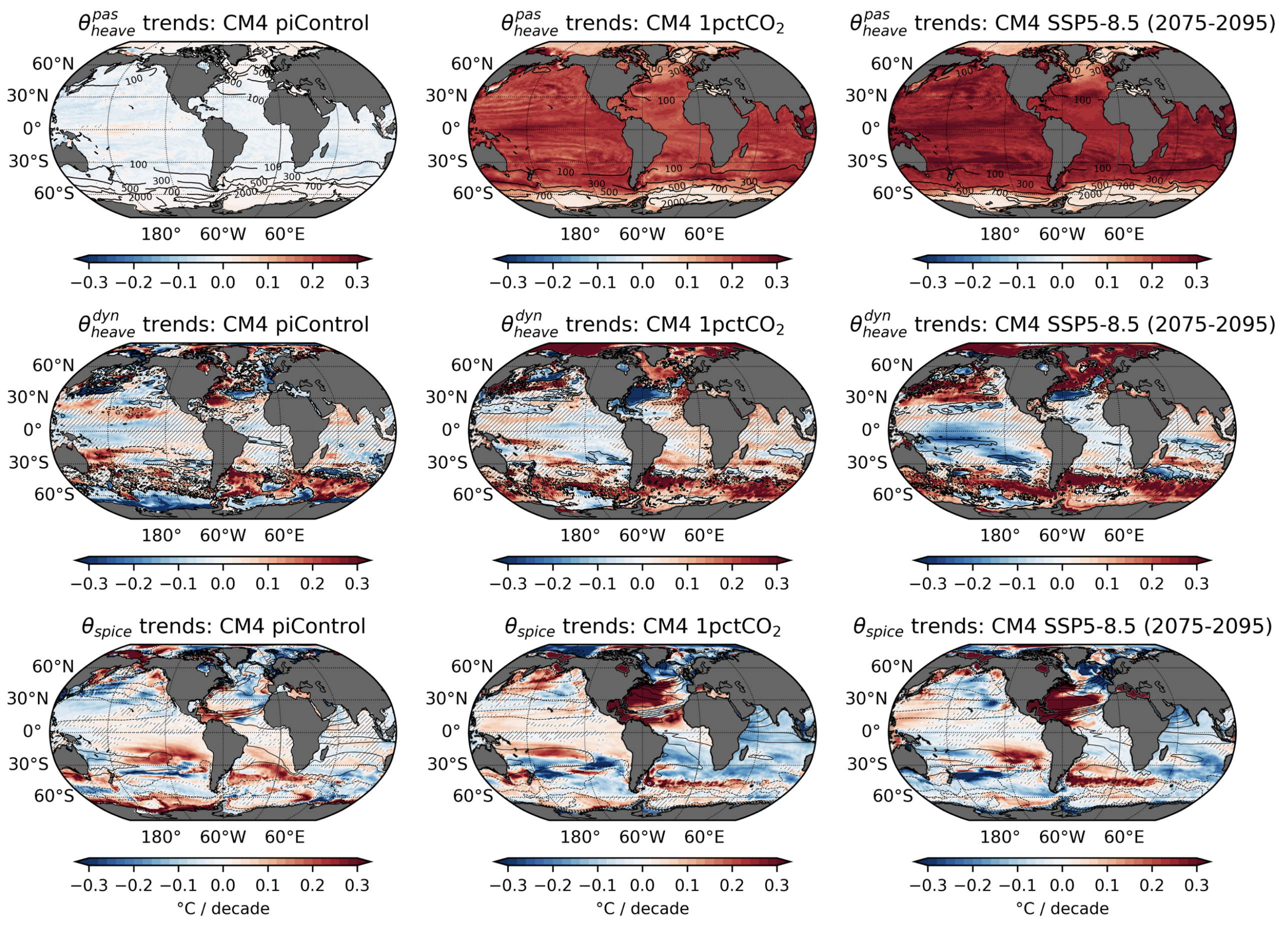

Supplementary Figure 3. Temperature trends attributed to (upper) the passive heave, (middle) dynamic heave and (lower) spice component, averaged over the 0-700m, derived from the CMIP6 piControl experiment (model years 630-650), 1pctCO_2 experiment (model years 130-150) and SSP5-8.5 scenario (calendar year 2075-2095) using the GFDL-CM4 model. Contours indicate (upper) the time-mean outcropping reference depth z_r (in meters), (middle) z_r trends of 5 and 10 m per decade and (lower) climatological spice levels ranging from from -5°C to 5°C in 1°C intervals, averaged over 0-700m. Hatching denotes regions where trends are not statistically significant at $p \leq 0.05$.
